# Ozone Treatment as an Approach to Induce Specialized Compounds in *Melissa officinalis* Plants

**DOI:** 10.3390/plants13070933

**Published:** 2024-03-23

**Authors:** Giulia Scimone, Maria Giovanna Carucci, Samuele Risoli, Claudia Pisuttu, Lorenzo Cotrozzi, Giacomo Lorenzini, Cristina Nali, Elisa Pellegrini, Maike Petersen

**Affiliations:** 1 Department of Agriculture, Food and Environment, University of Pisa, Via del Borghetto 80, 56124 Pisa, Italy; giulia.scimone@phd.unipi.it (G.S.); mariagiovanna.carucci@phd.studenti.it (M.G.C.); samuele.risoli@phd.unipi.it (S.R.); claudia.pisuttu@agr.unipi.it (C.P.); lorenzo.cotrozzi@unipi.it (L.C.); giacomo.lorenzini@unipi.it (G.L.); cristina.nali@unipi.it (C.N.); 2 University School for Advanced Studies IUSS Pavia, Piazza della Vittoria 15, 27100 Pavia, Italy; 3 Institut für Pharmazeutische Biologie und Biotechnologie, Philipps-Universität Marburg, Robert-Koch-Str. 4, D-35037 Marburg, Germany; petersen@mailer.uni-marburg.de

**Keywords:** secondary metabolites, lemon balm, bioactive properties, eustress, oxidative stress, rosmarinic acid, expression analysis

## Abstract

Plants are constantly subjected to environmental changes that deeply affect their metabolism, leading to the inhibition or synthesis of “specialized” compounds, small organic molecules that play a fundamental role in adaptative responses. In this work, *Melissa officinalis* L. (an aromatic plant broadly cultivated due to the large amounts of secondary metabolites) plants were exposed to realistic ozone (O_3_) dosages (80 ppb, 5 h day^−1^) for 35 consecutive days with the aim to evaluate its potential use as elicitor of specialized metabolite production. Ozone induced stomatal dysfunction throughout the whole experiment, associated with a low photosynthetic performance, a decrease in the potential energy conversion activity of PSII, and an alteration in the total chlorophyll content (−35, −36, −10, and −17% as average compared to the controls, respectively). The production of hydrogen peroxide at 7 days from the beginning of exposure (+47%) resulted in lipid peroxidation and visible injuries. This result suggests metabolic disturbance within the cell and a concomitant alteration in cell homeostasis, probably due to a limited activation of antioxidative mechanisms. Moderate accumulated doses of O_3_ triggered the accumulation of hydroxycinnamic acids and the up-regulation of the genes encoding enzymes involved in rosmarinic acid, phenylpropanoid, and flavonoid biosynthesis. While high accumulated doses of O_3_ significantly enhanced the content of hydroxybenzoic acid and flavanone glycosides. Our study shows that the application of O_3_ at the investigated concentration for a limited period (such as two/three weeks) may become a useful tool to stimulate bioactive compounds production in *M. officinalis*.

## 1. Introduction

Under ever-changing environmental conditions, plants have to deal with several abiotic and biotic adversities, which frequently have a negative impact on their productivity and growth [1]. For that reason, plants have evolved complex functional and genetic adaptation mechanisms that can be activated to counteract/cope with oxidative reactions, while continuing growth (even though at a decreased rate) and reaching a fruitful yield [2]. According to Munns and Millar [3], these mechanisms are related to taking up vital resources, while storing and supplying them to various plant parts, to produce the energy required for cellular functions, maintain tissue integrity, transmit signals among plant parts, arrange structural assets under changed environmental conditions, and shape-shift through development.

Abiotic stress factors often lead to oxidative damage by affecting the steady-state amount of carbohydrate structures, proteins, lipid membranes, and nucleic acids that are needed for the optimal function of plants. In addition, they potentially increase the degradation rate of some physiological and biochemical constituents, thus making necessary increased rates of repair [4]. Consequently, plant metabolism must be remarkably tuned to provide a valuable merging of a wide spectrum of metabolic pathways and results in antioxidant accumulation and redox signaling activation [5]. A pivotal component of the repair strategy is represented by “specialized” compounds: small organic molecules characterized by different molecular structures and several biological functions, which are classified in different categories, among which are phenylpropanoids, nitrogen-containing compounds, and terpenoids (based on their biosynthetic origin [6]). In particular, phenylpropanoid metabolism produces a high range of specialized compounds on the basis of a few intermediates of the shikimate pathway. The spectrum of hydroxycinnamic acids and esters is increased by various enzymatic conversions (by combining transferases, reductases, and oxygenases) to result in an organized and distinct set of species-specific metabolites [7]. These specialized secondary metabolites constitute an appreciable antioxidant system involved in defense mechanisms, especially in plant–environment interactions, by avoiding the production of reactive oxygen species (ROS) and quenching them once they are formed [8]. For this reason, functional and/or genetic alterations in these metabolites are generally a reliable feature in restoring redox homeostasis. If these responses are not occurring adequately, then the primary metabolism is altered and the oxidative burst becomes severe, subsequently leading to the death of cells and senescence [9]. Their success is related not only upon the huge numbers of metabolites synthesized by various taxa, but also on the multifunctionality of each specialized compound in relation to the type/intensity of stress factors, cultivar/provenance, and plant vegetative stage [10]. Consequently, the analysis of cellular, metabolic, and molecular rearrangements could elucidate the role(s) of specialized compounds in plant acclimation/adaptation to unfavorable conditions.

Hormesis is a dose-dependent response phenomenon where low doses of abiotic/biotic stress factors trigger defense mechanisms inducing benefits on plant performance, allowing them to remain “conditioned” in the face of future stress situations [11]. On the other hand, high doses of stress factors usually reduce/inhibit functional and genic processes by inducing uncertain outcomes on plant functioning and causing “distress” [12]. Elicitors (e.g., physical, chemical, and biological factors, which provoke unfavorable conditions, lead to defense mechanisms by producing specialized compounds, the activation of receptors/sensors, and switching on both the signaling pathways and the activity of specific enzymes against stress) can favor channeling plant energy towards the accumulation of specialized metabolites [13]. In particular, through the application of “beneficial stress” (eustress [14]) and the stimulation of a (moderate) stress response, a (over)-production of desired chemicals can be actively induced. For this reason, the use of abiotic/biotic elicitors could be regarded as an effective tool in the production of economically relevant antioxidant compounds, and a potential solution to the issues associated with their acquisition [15]. Therefore, elicitation (or priming) could be inserted into the plant cultivation protocols: firstly, plants could be grown under favorable environmental conditions and secondly, the production of specialized compounds could be induced by applying eustress in order to increase the quality of vegetables/foods and the related health benefits [14]. Other advantages of this phenomenon are related to its environmentally friendly nature, consumer acceptance, low costs, and application simplicity.

Plant exposure to a short time period of adequate ozone (O_3_) concentration, under controlled conditions, has been considered as a ready-to use tool to enhance their nutritional value, since it usually increases plant antioxidant compounds to counteract O_3_-induced oxidative pressure [16,17]. To date, O_3_ treatment has been used as a natural method for the purification/disinfection of wastewater, and the control of mycotoxigenic fungi and insects in stored products, due to its antimicrobial properties [18]. Ozone decomposes spontaneously in water into hydroxide radicals, hydrogen peroxide (H_2_O_2_), or oxygen by leaving no toxic residues. Consequently, the application of O_3_ in the food industry has been regarded as effective and safe by the World Health Organization, being recognized as a “green technology” for the treatment of vegetables, fruits, and grains [19]. The use of O_3_ as an elicitor for plant specialized metabolism may represent an important option for the pharmaceutical industry in order to develop nutraceuticals/novel drugs with a high safety, sustainability, and efficacy [20]. However, the response direction and magnitude of secondary metabolites (and specific classes) can differ depending on the (i) kinetics, dose of O_3_ application, (ii) plant genus/species/cultivar processed, (iii) developmental stage, and (iv) balance between avoidance and repair strategies in treated tissues [21]. To the best of our knowledge, few studies have examined the possible use of realistic O_3_ concentrations for a long time period to select a specific dose (based on plant hormesis) able to elicit the production of biochemicals and bioactive compounds that are essential sources of pharmaceuticals, flavors, and food additives.

A plant species that has shown an evident sensitivity to O_3_ (approached as a crucial environmental stress factor [22]) is *Melissa officinalis* (lemon balm, Lamiaceae). Native to the Mediterranean region and Central Asia, this herbaceous perennial plant is widely cultivated due to the high amounts essential oil and of phenolic metabolites contained in plant tissues and leaf essential oils such as hydroxycinnamic acid derivatives (rosmarinic acid represents the major constituent of this group), caffeic acid esters, and flavonoids (e.g., luteolin and apigenin for the flavones group, and hesperidin, hesperetin, and naringenin for the flavanones one), which play an active key role within lemon balm antioxidant mechanisms [23,24]. The interaction between *M. officinalis* and realistic/elevated O_3_ concentrations within a short time period (80 and 200 ppb for 5 h) from various perspectives has been one of the principal study objectives of our research group for about 15 years [25,26,27,28,29,30]. In particular, lemon balm sensitivity to O_3_ was well documented, in these investigations, in terms of photosynthetic performance, alteration in membranes, visible damage, crosstalk among hormones, signaling molecules and low-molecular-weight antioxidants, and changes in transcript abundance of several genes involved in the biosynthesis of rosmarinic acid (its main active phenolic compound). Overall, *M. officinalis* proved to be a model candidate in responding to a single pulse of O_3_. We demonstrated that an O_3_-avoidance strategy (i.e., the leaf’s capability to partially close stomata in to avoid entrance of O_3_ [31]) did not completely prevent the oxidative pressure and the early events of senescence [25,28]. At the same time, O_3_ repair strategies partially and transiently counteracted/reduced the occurrence of photo-oxidative stress and the cellular damage [26,27,29,30]. Here, *M. officinalis* plants were exposed to realistic O_3_ concentrations for a long time period [80 ppb for 35 consecutive days (5 h day^−1^)] in order to elucidate whether and how avoidance and/or repair strategies have been adopted by analyzing major markers for both qualitative and quantitative responses. We postulated that the rearrangement of the primary and specialized metabolism to arrange carbon sinks towards the specialized classes/compounds might represent a key element in plant elicitation.

## 2. Results

### 2.1. Visible Injury and Plant Biometric Traits

Starting from 14 days from the beginning of the exposure (FBE), plants showed the same symptoms already reported in Pellegrini et al. [29]: O_3_-treated plants presented a progressive presence of roundish and dark-blackish necrosis located in the interveinal adaxial areas of mature fully expanded leaves (see Figure S1). In the control and younger leaf material of O_3_-treated plants, no symptoms were detected. At the end of the exposure, the plant height, total fresh and dry weight, and total foliar area were slightly decreased by O_3_ (−6, −11, −17, and −35% compared with the control; Table 1). Conversely, the root to shoot ratio was significantly increased by O_3_ (+34%). No significant O_3_ effects were reported on the root length.

### 2.2. Leaf Ecophysiological Traits

**Figure 1:**
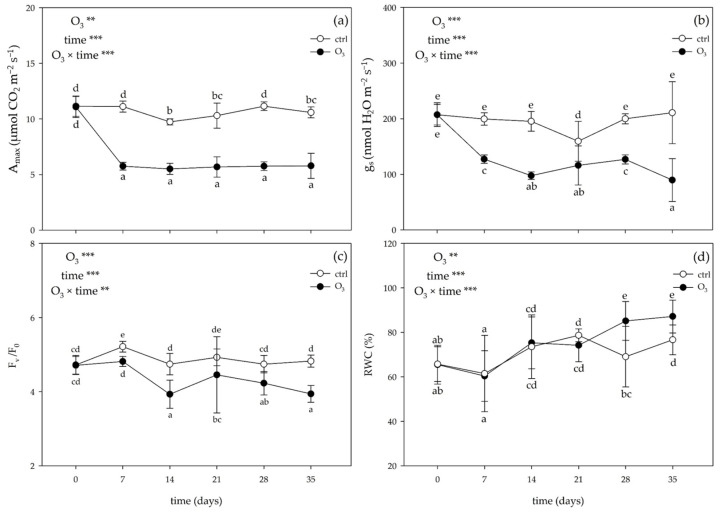
Variation in (**a**) photosynthetic activity at saturating light level (A_max_), (**b**) stomatal conductance (g_s_), (**c**) variable and minimal fluorescence ratio (F_v_/F_0_), and (**d**) relative water content (RWC) in leaves of *Melissa officinalis* plants exposed to charcoal-filtered air (open circle) or to 80 ppb of ozone (O_3_; 5 h day^−1^) for 35 consecutive days (closed circle). Data are shown as mean ± standard deviation (n = 5). For each parameter, *p* levels for the effects of the singular factor “O_3_” and “time”, and their combination from a one-way repeated measures ANOVA are shown (***: *p* ≤ 0.001; **: *p* ≤ 0.01). According to Tukey’s HSD post hoc test, different letters indicate significant differences among means.

The one-way repeated measures analysis of variance (ANOVA) of the gas exchange and chlorophyll *a* fluorescence parameters and the two-way ANOVA of the relative water content (RWC) values showed that the effects of the singular factor “O_3_” and “time”, and their combination were significant (Figure 1).

Ozone significantly decreased the photosynthetic activity at a saturating light level (A_max_) already at 7 days FBE (−48% compared with the control), and this effect was almost consistent until the end of the treatment (Figure 1a). Similarly, stomatal conductance (g_s_) was reduced by O_3_ from 7 days FBE (−36%) until the end of the treatment (−35%, as average; Figure 1b). Ozone decreased the maximum quantum efficiency of PSII photochemistry (F_v_/F_0_) starting from 7 days FBE (−8%), and this effect was almost consistent until the end of the treatment (Figure 1c). A marked reduction in the RWC values was reported at 28 and 35 days FBE (−19 and −12%, respectively; Figure 1d).

### 2.3. Leaf Biochemical Traits

#### 2.3.1. Hydrogen Peroxide Content and Oxidative Damage

**Figure 2:**
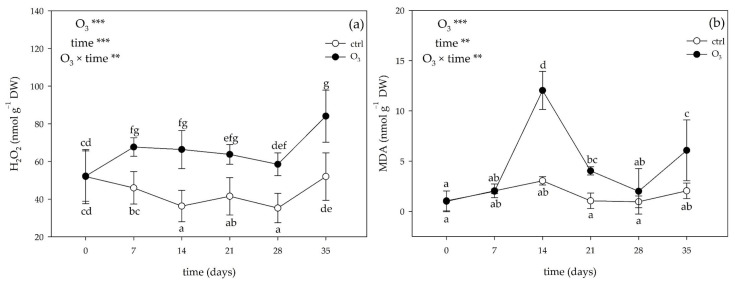
Variation in (**a**) hydrogen peroxide (H_2_O_2_) content, and (**b**) the concentration of malondialdehyde by-products (MDA) in leaves of *Melissa officinalis* plants exposed to charcoal-filtered air (control; open circle) or to 80 ppb of ozone (O_3_; 5 h day^─1^) for 35 consecutive days (closed circle). Data are shown as mean ± standard deviation (n = 5). For each parameter, *p* levels for the effects of the singular factor “O_3_” and “time”, and their combination from a two-way ANOVA are shown (***: *p* ≤ 0.001; **: *p* ≤ 0.01). According to Tukey’s HSD post hoc test, different letters indicate significant differences among means. Abbreviation: DW, dry weight.

The two-way ANOVA of hydrogen peroxide (H_2_O_2_) and malondialdehyde by-product (MDA) content showed that the effects of the singular factor “O_3_”and “time”, and their combination were significant (Figure 2). Ozone significantly increased the H_2_O_2_ concentration already at 7 days FBE (+47%), and this effect was almost consistent until the end of the exposure, reaching the highest value at 35 days FBE (+62%; Figure 2a). A variable O_3_ effect was instead reported for MDA levels: they did not show differences at 7 days FBE, significantly increased at 14 and 21 days FBE (4-fold than the control), came back to constitutive values at 28 days FBE, and increased again at the end of the exposure (3-fold than the control; Figure 2b).

#### 2.3.2. Photosynthetic Pigments and Low-Molecular-Weight Antioxidants

**Figure 3:**
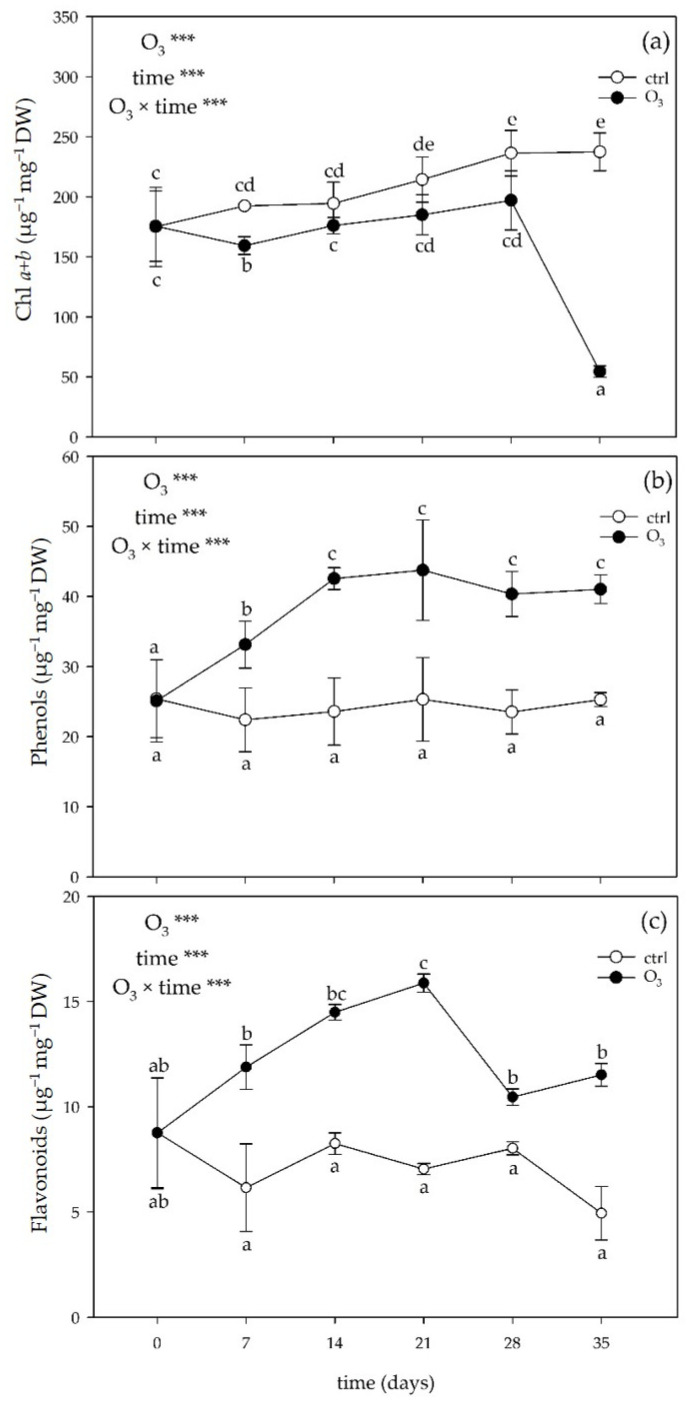
Variation in (**a**) total chlorophyll (Chl *a* + *b*), (**b**) phenols, and (**c**) flavonoids in leaves of *Melissa officinalis* plants exposed to charcoal-filtered air (control; open circle) or to 80 ppb of ozone (O_3_; 5 h day^─1^) for 35 consecutive days (closed circle). Data are shown as mean ± standard deviation (n = 5). For each parameter, *p* levels for the effects of the singular factor “O_3_” and “time”, and their combination from a two-way ANOVA are shown (***: *p* ≤ 0.001). According to Tukey’s HSD post hoc test, different letters indicate significant differences among means. Abbreviation: DW, dry weight.

The two-way ANOVA of the low-molecular-weight antioxidants showed that the effects of the singular factor “O_3_” and “time”, and their combination were significant (Figure 3). Ozone significantly decreased the content of chlorophyll (Chl) *a* + *b* at 7, 28, and 35 days FBE (−17, −17, and −77%, respectively; Figure 3a). No other significant effects were reported at other times of the analysis. In the treated leaves, the content of phenols was significantly higher than in controls already at 7 days FBE (+48%), and this effect was consistent until the end of the experiment (Figure 3b). Similarly, O_3_ also induced a significant accumulation of total flavonoids starting from 7 days FBE (+93%), and this effect lasted throughout the whole exposure (Figure 3c). At the end of the O_3_ treatment, the total carotenoids showed a significant decrease (−66% compared to the controls, 106 ± 30 vs. 36 ± 9 mg g^−1^ DW, *p* < 0.001).

**Figure 4:**
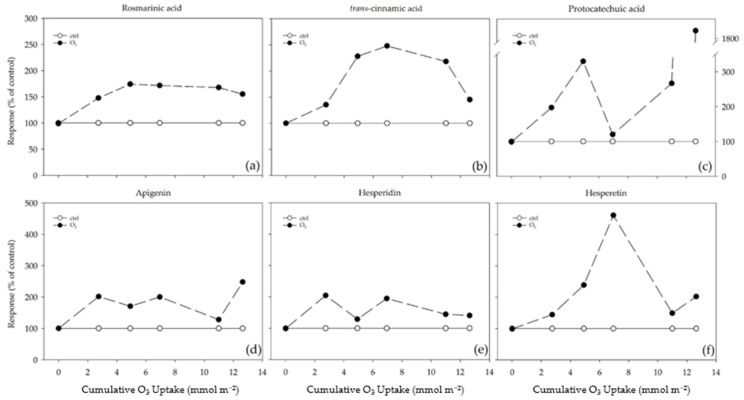
Responses of (**a**) rosmarinic acid, (**b**) *trans*-cinnamic acid, (**c**) protocatechuic acid, (**d**) apigenin, (**e**) hesperidin, and (**f**) hesperetin over a range of cumulative ozone (O_3_) uptakes (CUOs) in leaves of *Melissa officinalis* plants exposed to charcoal-filtered air (control = 100%; open circle) or to 80 ppb of O_3_ (5 h day^−1^) for 35 consecutive days (closed circle). The responses were calculated as Response = *µ_C_*/*µ_T_* × 100, where *µ_C_* is the mean value of *μ* of the control and *µ_T_* is the mean value of *μ* of the O_3_-treated samples.

A total of six phenylpropanoid compounds (see Figure S2) were identified in lemon balm leaves: two hydroxycinnamic acids (rosmarinic and *trans*-cinnamic acid), one hydroxybenzoic acid (protocatechuic acid), one flavone (apigenin), and two flavanone glycosides (hesperidin and hesperetin), respectively. The highest concentrations were detected for rosmarinic acid, which reached around 70% of the total of all phenolic compounds analyzed. The effect of O_3_ on these compounds is individually shown as percent changes over a range of cumulative O_3_ uptakes (CUOs) in Figure 4. In the leaves of the O_3_-treated plants, rosmarinic acid increased, starting from a CUO of 2.75 mmol m^−2^ (+48% compared to the control), and this effect lasted throughout the experiment (Figure 4a). The effect of O_3_ also increased the content of *trans*-cinnamic acid, which reached a peak at a CUO of 6.95 mmol m^−2^ (more than 2-foldthan the control; Figure 4b). In the case of both hydroxycinnamic acids, a slight decrease was observed at the end of the experiment. Conversely, the protocatechuic acid content did not exhibit a clear trend: it increased already at a CUO of 2.75 mmol m^−2^ (+97%) before reaching a maximum at a CUO of 4.93 mmol m^−2^, came back to the control level at a CUO of 6.95 mmol m^−2^, and increased again until the end of the exposure (more than 18-fold than the control; Figure 4c). Similarly, the apigenin content did not exhibit a clear trend: it increased at a CUO of 2.75 mmol m^−2^ (2-fold than the control; Figure 4d), slightly decreased at a CUO of 4.93 mmol m^−2^, increased at a CUO of 6.95 mmol m^−2^, came back to the control level at a CUO of 10.99 mmol m^−2^, and increased again at the end of the experiment. The hesperidin and hesperetin contents increased in the treated leaves already at a CUO of 2.75 mmol m^−2^ (2-fold and +44%, respectively, in comparison to the control; Figure 4e,f) before reaching a maximum at 2.75 and 6.95 mmol m^−2^, decreasing at a CUO of 4.93 and 10.99 mmol m^−2^, before increasing again in the remaining times of the analysis.

#### 2.3.3. Activity of Phenylalanine Ammonia-Lyase

**Figure 5:**
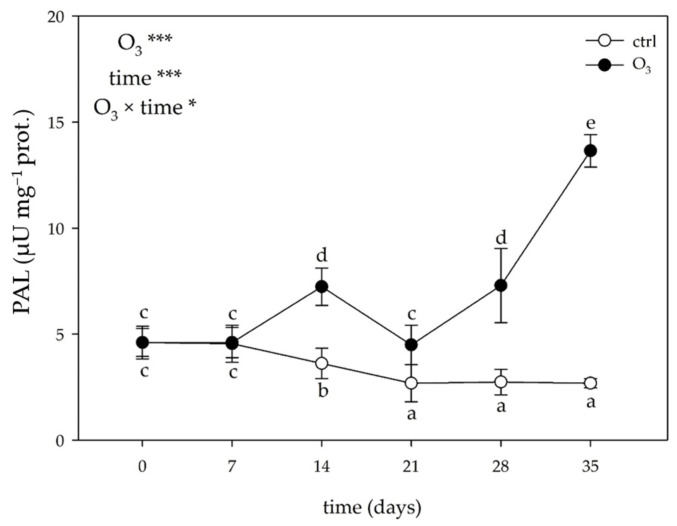
Variation in phenylalanine ammonia-lyase (PAL) activity in leaves of *Melissa officinalis* plants exposed to charcoal-filtered air (open circle) or to 80 ppb of ozone (O_3_, 5 h day^─1^) for 35 consecutive days (closed circle). Data are shown as mean ± standard deviation (n = 5). *p* levels for the effects of the singular factor “O_3_” and “time”, and their combination from a two-way ANOVA are shown (***: *p* ≤ 0.001; *: *p* ≤ 0.05). According to Tukey’s HSD post hoc test, different letters indicate significant differences among means. One unit of PAL activity was defined as the amount of enzyme required to produce 1 μmol of *trans*-cinnamic acid per min.

The two-way ANOVA of the phenylalanine ammonia-lyase (PAL) activity showed that the effects of the singular factor “O_3_”and “time”, and their combination were significant (Figure 5). Ozone significantly increased the activity of PAL at 14 days FBE (2-foldthan the controls) and reached a peak at 35 days FBE (5-foldthan the controls).

#### 2.3.4. Total Antioxidant Activity

**Figure 6:**
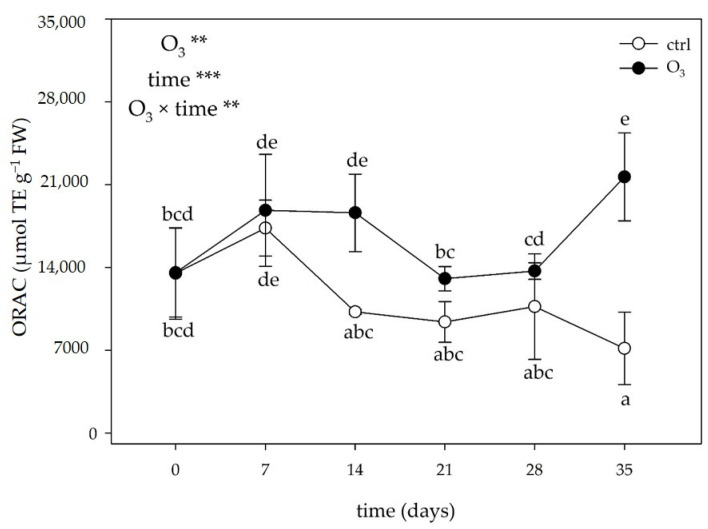
Variation in oxygen radical absorption capacity (ORAC) in leaves of *Melissa officinalis* plants exposed to charcoal-filtered air (open circle) or to 80 ppb of ozone (O_3_, 5 h day^─1^) for 35 consecutive days (closed circle). Data are shown as mean ± standard deviation (n = 5)., *p* levels for the effects the effects of the singular factor “O_3_” and “time”, and their combination from a two-way ANOVA are shown (***: *p* ≤ 0.001; **: *p* ≤ 0.01). According to Tukey’s HSD post hoc test, different letters indicate significant differences among means. Abbreviation: TE, trolox equivalent.

The two-way ANOVA of the total antioxidant activity showed that the effects of the singular factor “O_3_”and “time”, and their combination were significant only for the oxygen radical absorption capacity (ORAC; Figure 6). In the treated leaves, O_3_ induced a significant increase in the antioxidant activity at 14 and 35 days FBE (+82% and 3-foldin comparison to the control, respectively). No other significant effects were observed at other times of the analysis.

### 2.4. Leaf Molecular Traits

**Figure 7:**
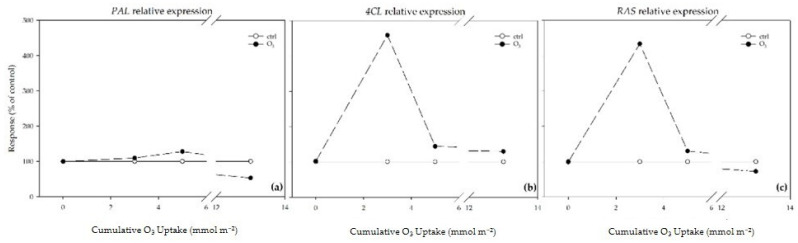
Responses of (**a**) phenylalanine ammonia-lyase (*PAL*), (**b**) 4-coumarate CoA-ligase (*4CL*), and (**c**) rosmarinic acid synthase (*RAS*) relative expression, over a range of cumulative ozone (O_3_) uptakes (CUOs) in leaves of *Melissa officinalis* plants exposed to charcoal-filtered air (control = 100%; open circle) or to 80 ppb of O_3_ (5 h day^−1^) for 35 consecutive days (closed circle). The responses were calculated as response = *µ_C_*/*µ_T_* × 100, where *µ_C_* is the mean value of *μ* of the control and *µ_T_* is the mean value of *μ* of the O_3_-treated samples.

The effect of O_3_ on the transcript abundance of *PAL*, 4-coumarate CoA-ligase (*4CL*), and rosmarinic acid synthase (*RAS*) is individually shown as percent changes over a range of CUOs in Figure 7. In the treated leaves, the *PAL* relative expression was slightly up-regulated at a CUO of 2.75 and 4.93 mmol m^−2^ (+10 and +28%, respectively, compared to the control plants), and then was down-regulated at the end of the experiment (−47%; Figure 7a). The effect of O_3_ also induced an up-regulation of the *4CL* and *RAS* transcripts, which reached a peak at a CUO of 2.75 mmol m^−2^ (more than 4- and 5-fold higher than the controls, respectively; Figure 7b,c). A down-regulation of the *RAS* transcripts was observed at the end of the experiment (−28%; Figure 7c).

## 3. Discussion

Throughout human history, plant specialized metabolites have always represented an important source of bioactive ingredients to exploit in various sectors. In particular, specialized compounds contained in both cultivated aromatic and medicinal plants proportionally assign them an important economic value: their extraction is fundamental for commercial pharmaceuticals, cosmetics, nutraceuticals, and food additives [32]. Unfortunately, when growing in the optimal environment, the above-mentioned plants do not always reach the amount required for meeting large-scale production [33]. Thus, industry is now focusing on alternative ways that can easily increase specialized metabolite accumulation. Across all the various technologies that have already been studied in the research field (i.e., abiotic stressors such as light, salinity, drought), O_3_ was always treated as one of the top trending topics [34]. Its important stimulatory role within the antioxidant system was elucidated through the years in many works [35], but few regarded the consequence of O_3_ exposure at environmental concentrations for a prolonged time. In these terms, induced hormesis could represent a valuable instrument for effectively controlling and increasing the content of those compounds [36]. The aim of this study was to prove that an adequate O_3_exposure can act as a simple, green, and low-cost elicitor for specialized metabolites in lemon balm plants.

At the end of the experiment, *M. officinalis* leaves were injured, their growth was markedly inhibited, and their ability to produce photosynthates was limited. The observed imbalance between epigeous and hypogeous growth (i.e., increased root/shoot ratio) could be due to the following: (i) changes in carbon dynamics into plants, (ii) impairment of phloem function/structure, (iii) investment in antioxidant compounds, and (iv) alteration in photosynthetic carbon gain by stomatal closure [11]. This phenomenon can have two interpretations: avoidance or sluggishness (e.g., the loss of stomatal control that may eventually cause a reduction in carbon and water-use efficiency; [37]). Ozone induced stomatal dysfunction throughout the whole experiment, which was associated with a low photosynthetic performance (e.g., decreased A_max_ values), a decrease in the potential energy conversion activity of PSII (e.g., decreased F_v_/F_0_ ratio), and a decrease in the total Chl content (observed at 7, 28, and 35 days FBE). This reduction seems to be a first attempt to avoid the inhibition and partial maintenance of leaf water content (as indicated by the unchanged RWC values; [38]), and a subsequent O_3_-induced damage of subsidiary cells, when direct or indirect Chl breakdown occurred [39]. The induction of an O_3_ oxidative burst induction (i.e., a marked over-production of H_2_O_2_) not only affects chloroplast components but also cell wall and plasma membrane ones, which resulted in lipid peroxidation (as confirmed by the increase in the MDA content at 14, 21, and 35 days FBE), and visible injuries [40,41]. This result suggests metabolic disturbance within the cell and a concomitant alteration in cell homeostasis, probably due to an inadequate response of the antioxidative systems.

Plant tolerance to oxidative stress also depends on biochemical components, such as the capacity of leaves to activate detoxifying mechanisms [41]. It is known that specialized metabolites are well suited to constitute a “secondary” antioxidant system with a pivotal role in plant defense against abiotic and biotic stress factors [42]. Changes in phenylpropanoid compounds can be regarded as a repair strategy that can equip stressed plants with an additional antioxidant system capable of scavenging/avoiding ROS and preventing water loss [7]. In addition, carotenoids can transiently complement their action by dissipating excess excitation energy or suppressing lipid peroxidation [43]. In our study, realistic O_3_ treatment resulted in an overall accumulation of total phenols and total flavonoids, indicating a rearrangement of the phenylpropanoid pathway with various functions in order to generate carbon intermediates and support detoxification mechanisms [44,45]. The concomitant increase of PAL activity (which was selected to be studied among other key secondary metabolic enzymes due to its fundamental role as entry-point enzyme of the general phenylpropanoid pathway [46]), starting from 14 days FBE, confirms the shift from primary metabolism towards phenols and flavonoids in order to provide antioxidative protection to chloroplasts and alleviate the excess of excitation pressure [47]. The reduction in the total carotenoids observed at the end of the experiment suggests that these molecules could be consumed by the cell to counteract the increased oxidative pressure, potentially working as photoprotectors [48]. However, these additional antioxidative mechanisms were not able to repair the cell structure and prevent cellular damage. Even though no clear relationship between the activation of antioxidant compounds and stress tolerance was established, it is demonstrated that the biosynthesis of phenylpropanoids and carotenoids increases more in stress-sensitive species such as *Melissa officinalis* than in tolerant ones (e.g., *Salvia officinalis*) [41].

Considering phenylpropanoid compounds are able to act as antioxidants in a variety of ways, as well as their multiple functions in leaves, the documented induction of specific classes/metabolites at specific times of analysis can be regarded as hormesis [49]. It can be hypothesized that plants accumulated phenylpropanoids at low/moderate O_3_ dosages as an induced defense against oxidative burst, while the production of these compounds was reduced at higher O_3_ levels due to lipid peroxidation and cell damage that occur when the defense responses are overwhelmed [50]. Here, *M. officinalis* leaves did not exhibit monotonic/linear dose–time relationships to O_3_ treatment by showing chemical composition plasticity against oxidative stress. A biphasic exposure–response curve was observed in the case of rosmarinic and *trans*-cinnamic acids: their concentrations initially increased (with a maximum value in the moderate-dose zone; phase I), then decreased as the O_3_ exposure increased (phase II). This result indicates that reaching too high doses of O_3_ resulted in a slow inhibition of the hydroxycinnamic acid pathways. In particular, the mechanisms regulating genes encoding enzymes involved in phenylpropanoid biosynthesis were (in part) at the transcriptional level by reflecting the sequence of events taking place throughout the whole experiment [51]. In fact, a biphasic exposure–response curve was also observed in the case of the *PAL*, *C4L,* and *RAS* transcripts: their concentrations initially increased [with a maximum value in the low (observed for *4CL* and *RAS*) and moderate-dose zone (observed for *PAL*); phase I], then decreased as the O_3_ exposure increased (phase II). It is possible to speculate that increasing the O_3_ concentrations could affect the enzyme integrity and the tonoplast by resulting in metabolic disturbances (especially in the case of rosmarinic acid, which is compartmentalized to vacuoles; [52]), destruction of the cell’s compartmentalization, cell death, and visible injuries [53]. A biphasic exposure–response curve (with 1 and 2 points of inflexion) was also reported in the case of flavanone glycosides. In particular, the levels of hesperetin initially increased (before reaching the maximum value in the moderate-dose zone; phase I), then decreased as the O_3_ exposure increased (phase II), and slightly increased again (phase III). A similar trend was observed for the hesperidin values: phase I peaked in the low-dose zone and phase III at the moderate/high-dose one. This result indicates that to regulate the cellular redox state, lemon balm plants transiently lost their ability in investing into the flavanone glycoside pathway by diversifying the components of the phenylpropanoid compounds available [54]. Conversely, protocatechuic acid and apigenin exhibited a biphasic exposure–response curve with a stimulatory effect: in particular, their levels initially increased (phase I), then decreased (before reaching the control values in the case of protocatechiuc acid; phase II), and markedly increased again as the O_3_ exposure increased (with a maximum value in the high-dose zone, phase III). The increasing production of both hydroxybenzoic acid and flavones potentially suggests O_3-_accumulated doses may be effective for priming purposes [14]. Interestingly, this activation was accompanied by an increase in the total antioxidant activity (expressed as the ORAC values), which was also enhanced at 14 and 35 days FBE, suggesting that these metabolites play a role as “primary” oxidants (as scavengers of peroxyl radicals) and/or “secondary” antioxidants (e.g., indirect pathway) against chronic O_3_ exposure [50]. In particular, it is possible to conclude that the capability of *M. officinalis* to regenerate active reduced forms and prevent radical formation was finely regulated by the activation/suppression of specific classes/metabolites and the up- and down-regulation of the transcript levels of genes encoding key biosynthetic enzymes at specific times of the analysis [41].

In conclusion, the regulatory responses of *M. officinalis* to moderate/realistic O_3_ concentrations for a long time period showed that this species reacts to O_3_ depending on the duration and the intensity of the treatment. Moderate doses of O_3_ triggered the accumulation of hydroxycinnamic acids and the up-regulation of the genes encoding enzymes involved in rosmarinic acid, phenylpropanoid, and flavonoid biosynthesis. While high doses of O_3_ significantly enhanced the content of hydroxybenzoic acid and flavanone glycosides. However, these changes coincided with an impairment of the photosynthetic performance and oxidative damage, which may indicate the involvement of ROS imbalance in the stimulation of plant specialized metabolism. Our study shows that the application of O_3_ at the investigated concentration for a limited period (such as two/three weeks) may become a valuable biotechnological tool to induce the production of bioactive compounds in *M. officinalis*.

## 4. Materials and Methods

### 4.1. Plant Material and Experimental Design

Experiments were conducted at the field station of the Department of Agriculture, Food and Environment of the University of Pisa, located in San Piero a Grado (Pisa, Italy; 43°40′48″ N, 10°20′48″ E, 2 m a.s.l.). Four-month-old cuttings of *M. officinalis*, purchased from a local nursery, were transplanted to plastic pots containing a mix of steam-sterilized soil and peat (1:1, in volume) and collocated for two weeks in a facility under controlled environmental conditions. Uniformly-sized plants (ca. 40 cm of height) were placed in four fumigation chambers under the same environmental conditions as reported above, equally divided into two groups and exposed to O_3_-free charcoal-filtered air (controls) or to a target O_3_ concentration of 80 ppb (1 ppb = 1.96 μg m^−3^, at 25 °C and 101.325 kPa) for 35 consecutive days (5 h day^−1^, in the form of a square wave from 10:00 a.m. to 03:00 p.m.; see Figure S3). Further details about the experimental conditions and methodology are reported in Marchica et al. [14]. Analyses were carried out at 7, 14, 21, 28, and 35 days FBE, corresponding to CUOs of 2.75, 4.93, 6.95, 10.99, and 12.64 mmol m^−2^, respectively. The CUO was calculated according to Pellegrini [55], by applying the equation CUO (mmol m^−2^) = CEO_3_ g_s_ kO_3_ 3600 × 10^−6^, where kO_3_ = 1.67 is the ratio of the leaf resistance to water, g_s_ is the leaf-level stomatal resistance (in units of mol H_2_O m^−2^ s^−1^), and CEO_3_ is the cumulative exposure to O_3_ calculated as follows: CEO_3_ (nmol mol^−1^ h) = [O_3_] H D, where H is the number of daytime hours in which the plant was exposed to O_3_, D is the total number of days, and [O_3_] is the external O_3_ concentration (in ppb) that plants were exposed to during daytime hours of the whole experimental period. At each time of analysis, at least five plants at the vegetative stage per O_3_ treatment were randomly selected and measured in terms of gas exchange and chlorophyll *a* fluorescence. Then, using other plants at the vegetative stage, fully expanded leaves (equally distributed over plant height) per plant were sampled, immediately frozen in liquid nitrogen, grounded and stored at −80 °C until biochemical and molecular analyses. Thirteen plants were randomly selected by using a software function (each plant was numbered and casually attributed to a treatment) at the end of the experiment and devoted to the biomass assessments. The onset and the development of symptoms were monitored throughout the whole experiment.

### 4.2. Plant Biometric Traits

After harvesting, organs (leaves, stems, and roots) were properly separated, washed in order to remove soil particles, weighed, and maintained at 60 °C until constant weight.

### 4.3. Leaf Physiological Traits

Photosynthetic activity at saturating light level and g_s_ were measured by using a portable photosynthesis system (Li-COR, Model 6400, Lincoln, NE, USA), operating at an ambient CO_2_ concentration (400–420 ppm), and 1500 μmol photons m^−2^ s ^−1^. Chlorophyll *a* fluorescence measurements were performed using a PAM-2000 fluorometer (Walz, Effeltrich, Germany) according to Marchica et al. [51]. Relative water content was determined according to Penella et al. [38], using the following equations: 100 × (FW − DW)/(TW − DW), where FW is the fresh weight, TW is the turgid weight after rehydrating samples for 24 h in distilled water, DW is the dry weight after oven-drying samples at 80 °C for 72 h. Single leaf area (n = 13) was measured using ImageJ software (version 1.52t).

### 4.4. Leaf Biochemical Traits

#### 4.4.1. Assessment of Hydrogen Peroxide Content and Membrane Damage

Leaf H_2_O_2_ production was measured fluorometrically by using the Amplex Red Hydrogen Peroxide/Peroxidase Assay Kit (Molecular Probes, Invitrogen, Carlsbad, CA, USA), according to Shin et al. [56]. Leaf material (0.06 g FW) was added to 1 mL 20 mM potassium phosphate buffer (pH 6.5). After centrifugation at 13,000× *g* for 10 min at 4 °C, the supernatant was incubated with a mixture of 10-acetyl-3,7-dihydrophenoxazine (10 mM), horseradish peroxidase (10 U mL^−1^), and potassium phosphate buffer (20 mM, pH 6.5). Samples were incubated for 30 min at 25 °C in the dark and the resorufin fluorescence (*E_x_*/*E_m_* = 530/590 nm) was quantified with a fluorescence/absorbance microplate reader (Victor3 1420 Multilabel Counter, Perkin Elmer, Waltham, MA, USA), after subtracting the background fluorescence due to the buffer solution and to the assay reagents.

Lipid peroxidation was estimated by determining the MDA accumulation according to the method of Hodges et al. [57], with minor modifications. Leaf material (1 g FW) was added to 2.5 mL 0.1% (*w*/*v*) trichloroacetic acid (TCA). The supernatant was mixed with 4 mL 20% (*w*/*v*) TCA and 0.025 mL 0.5% (*w*/*v*) thiobarbituric acid (TBA). The mixture was heated at 95 °C for 30 min, immediately cooled, and centrifuged at 13,000× *g* for 30 min at 4 °C. The supernatant was used to determine the MDA concentration at 532 nm and corrected for non-specific turbidity by subtracting the absorbance at 440 and 600 nm, by using the fluorescence/absorbance microplate reader previously reported. In addition, to not overestimate the MDA values with other compounds detectable at 532 nm, sub-samples were determined without TBA, and their absorbance was subtracted from the value of the TBA-containing sample.

#### 4.4.2. Determination of Photosynthetic Pigments and Phenylpropanoids

Photosynthetic pigments were determined by ultra-high-pressure liquid chromatography (UHPLC) using a UHPLC Dionex UltiMate 3000 system equipped with an Acclaim 120 C18 column (5 µm particle size, 4.6 mm internal diameter × 150 mm length) maintained in a Dionex TCC-100 column oven at 30 °C, and a Dionex UVD 170U UV-Vis detector (Thermo Scientific, Waltham, MA, USA), according to Marchica et al. [51]. Leaf material (0.03 g FW) was homogenized in 3 mL 100% HPLC-grade methanol overnight. The supernatant was filtered through 0.2 μm Minisart SRT 15 filters, and immediately analyzed. The pigments were eluted using 100% solvent A (acetonitrile/methanol, 75:25, *v*/*v*) for the first 14 min, followed by a 1.5 min linear gradient to 100% solvent B (methanol/ethylacetate, 68:32, *v*/*v*), 15 min with 100% solvent B, which was pumped for 14.5 min, followed by 2 min linear gradient to 100% solvent A. The flow rate was 1 mL min^−1^. The pigments were detected by their absorbance at 445 nm. The sum of neoxanthin, violaxanthin, antheraxanthin, lutein, zeaxanthin, and β-carotene was considered as a measure of the total carotenoid content. Phenylpropanoids were analyzed by UHPLC as previously reported according to Marchica et al. [51]. Leaf material (0.03 g FW) was extracted in 3 mL of a mixture of 100% HPLC-grade methanol, H_2_O, and HCl (84:15:1 *v*/*v*/*v*) overnight at room temperature. After centrifugation at 13,000× *g* for 10 min at 4 °C The supernatant was filtered through 0.2 μm Minisart SRT 15 filters and immediately analyzed. The separation was performed at 30 °C and phenolic compounds were eluted using 2 min linear gradient from 100% solvent A (H_2_O/acetic acid, 99:1, *v*/*v*) to 5% solvent B (methanol/acetonitrile/acetic acid, 94:5:1, *v*/*v*/*v*), 8 min linear gradient to 25% solvent B, followed by 10 min linear gradient to 45% solvent B, 10 min linear gradient to 50% solvent B, followed by 20 min linear gradient to 100% solvent B, 5 min isocratic flow of 100% solvent B, and 15 min linear gradient from 100% to 5% solvent B. The flow rate was 1.5 mL min^−1^. Phenylpropanoids were detected by their absorbance at 280 nm. To quantify the pigment and phenylpropanoid content, known amounts of pure standard were injected into the UHPLC system and an equation, which correlates the peak area to pigment concentration, was formulated. Chromatographic data were processed and recorded by the Chromeleon Chromatography Management System software, version 6.60-2004 (Thermo Scientific).

#### 4.4.3. Activity of Phenylalanine Ammonia-Lyase

The activity of PAL (EC 4.3.1.24) was evaluated by measuring the *trans*-cinnamic acid produced from L-phenylalanine, according to Cotrozzi et al. [45]. Leaf material (0.3 g FW) was homogenized in 3 mL of a buffer containing 100 mM potassium borate (pH 8.8) with 14 mM 2-mercaptoethanol. The homogenate was then centrifuged at 13,000× *g* for 30 min at 4 °C. The PAL assay was carried out by using a reaction mixture [2% (*w*/*v*) L-phenylalanine in 100 mM potassium borate (pH 8.8) and enzyme extract]. After incubation for 120 min at 37 °C, the reaction was stopped by adding 50 μL 6 N HCl. The *trans*-cinnamic acid produced was measured at 290 nm by using a UV-Vis 1900 spectrophotometer (Shimadzu, Kyoto, Japan). The determination of proteins was carried out according to Bensadoun and Weinstein [58] by using bovine serum albumin as standard.

#### 4.4.4. Total Antioxidant Activity

The antioxidant activity was tested by measuring the hydroxyl radical antioxidant capacity (HORAC) and ORAC of the leaf methanol extracts using the OxiSelect HORAC and ORAC Activity Assay (Cell Biolabs, San Diego, CA, USA) according to Ou et al. [59,60]. The antioxidant activity was quantified at 480 and 530 nm (excitation and emission) with the microplate reader previously reported and expressed in terms of gallic acid for HORAC and Trolox equivalents (TE) for ORAC.

### 4.5. Leaf Molecular Traits

#### 4.5.1. RNA Isolation and First Strand cDNA Synthesis

Total RNA was extracted from frozen (−80 °C) leaf tissue by using β-mercaptoethanol and phenol for the extraction, and isopropanol for the precipitation according to the method reported by Giuliano et al. [61]. The quality of RNA was assessed by separation on a 0.7% (*w*/*v*) agarose gel run in 0.5 × Tris borate-EDTA buffer at 80 V and visualized by SYBR Green I nucleic acid gel stain. Complementary DNA (cDNA) was prepared from 1 μg of total RNA using oligo (dT) primers and Superscript reverse transcriptase (Invitrogen, Carlsbad, CA, USA), following the manufacturer’s instructions.

#### 4.5.2. Expression Analysis

For semi-quantitative reverse transcription PCR (RT-PCR) of *PAL*, *4CL,* and *RAS* sequences, degenerate oligonucleotide primers designed to conserved amino acid regions (with an amplicon length of ~300 bp; Table 2) were used. All degenerate oligonucleotide primers were synthesized by Eurofins MWG Operon (Ebersberg, Germany). PCR products were amplified from 250 ng of first-strand cDNA using the appropriate primers and Taq DNA polymerase (Hybaid, Ashford, UK). Reactions to amplify *PAL*, *4CL,* and *RAS* sequences were cycled at 94 °C for 30 s, 54 (for *RAS*), 57 (for *PAL*), and 65 °C (for *4CL*) for 1 min, and 70 °C for 2 min (30 cycles). The amplification of *β-actin* was used as the endogenous control, which was expected to show a constitutive expression pattern. Three technical replicates were analyzed for each biological sample per treatment. Amplification products were separated in an agarose gel run (as reported above), and the image of each agarose gel was captured by using a digital camera. Gene expression was normalized to the housekeeping *β-actin* band density.

### 4.6. Statistical Analysis

Normal distribution of data was analyzed by the Shapiro–Wilk *W* test. The effect of O_3_, time, and their interaction was investigated using a two-way ANOVA. Comparisons among means were determined by Tukey’s HSD post hoc test. For analyses carried out at the end of the experiment (biometric parameters and total content of carotenoids), the effects of O_3_ were analyzed by the Student’s *t*-test. Statistical analyses were performed in JMP^®^ Pro 14.0 (SAS Institute Inc., Cary, NC, USA).

## Figures and Tables

**Table 1:** Biometric traits in *Melissa officinalis* plants exposed to charcoal-filtered air (control) or 80 ppb of ozone (O_3_; 5 h day^−1^) for 35 consecutive days (O_3_). Data are shown as mean ± standard deviation (n = 13). For each parameter, the effect of O_3_ was tested by the Student’s *t*-test (***: *p* ≤ 0.001; **: *p* ≤ 0.01; *: *p* ≤ 0.05). Abbreviations: DW, dry weight; FW, fresh weight.

	Control	O_3_	*p*
Height (cm)	39.1 ± 3.23	36.8 ± 2.64	*
Total FW (g)	115.4 ± 13.06	102.4 ± 10.26	*
Total DW (g)	13.1 ± 2.07	10.9 ± 1.51	**
Total foliar area (cm^2^)	3769 ± 1037.5	2470 ± 301.8	***
Root to shoot ratio	0.32 ± 0.054	0.43 ± 0.059	**

**Table 2:** Degenerate primers for housekeeping gene (β-actin) and specific primers of *Melissa officinalis*, where Y = T, A; and R = A, T. The amplicon length of approximately 300 bp.

Primers	Sequences
*β-Actin*	F: 5-GGATGATATGGAGAAGATYTGTGGC-3′
	R: 5′-AGATCACGMCCAGCRAGATC-3′
*PAL*	F: 5′-ATACATATGGCAGAGAACGGTCATCATGATTCC-3′
	R: 5′-ATACATATGCTAGCAGATAGGCAGAGGTCCACCATT-3′
*4CL*	F: 5′-ATGGAGAACCCGGCAGGCCAAG-3′
	R: 5-GACTGCAGCTGCTAATCTTGATCT-3′
*RAS*	F: 5′ATGAGGATCGATATCAAGGAC-3′
	R: 5′TCAAATCTCATAAAACAACTTCTCAA-3′

## Data Availability

Data are contained within the article and Supplementary Materials.

## Supplementary Materials

The following supporting information can be downloaded at: https://www.mdpi.com/article/10.3390/plants13070933/s1.
