# Supplementary material for: Ozone Treatment as an Approach to Induce Specialized Compounds in Melissa officinalis Plants
Source: Plants (Basel). 2024 Mar 23;13(7):933. doi: 10.3390/plants13070933 (PMC11013203; doi:10.3390/plants13070933)
Supplement: Supplementary file 1 [file plants-13-00933-s001.zip › plants-2903181-supplementary-done.pdf]

plants

Article

Ozone Treatment as an Approach to Induce "Specialized" Compounds in *Melissa officinalis* Plants

Giulia Scimone, Maria Giovanna Carucci, Samuele Risoli, Claudia Pisuttu, Lorenzo Cotrozzi, Giacomo Lorenzini, Cristina Nali, Elisa Pellegrini, Maike Petersen

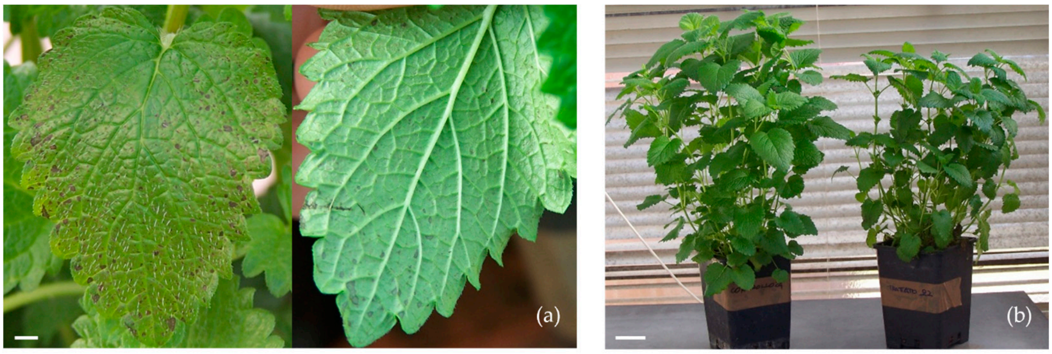

Figure S1. (a) Leaf symptoms on *Melissa officinalis* plants exposed to 80 ppb of O_3 (5 h day^{-1}) after 14 days from the beginning of the exposure (bar 0.4 cm). **(b)** Plants of *Melissa officinalis* exposed to charcoal-filtered air (on the left side) or 80 ppb of ozone (5 h day^{-1}) for 35 consecutive days (on the right side; bar 5 cm).

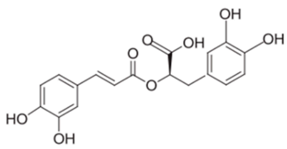

Rosmarinic acid

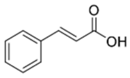

trans-cinnamic acid

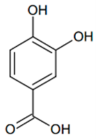

Protocatechuic acid

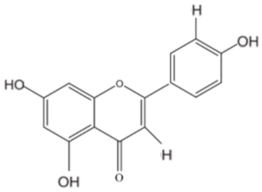

Apigenin

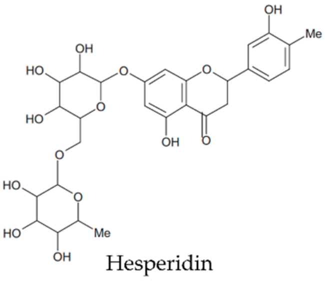

Hesperidin

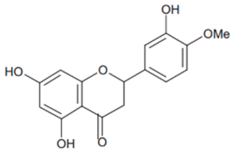

Hesperetin

Figure S2. Chemical structures of measured phenylpropanoid compounds (rosmarinic acid, trans-cinnamic acid, protocatechuic acid, apigenin, hesperidin and hesperetin) extracted from *Melissa officinalis* leaves.

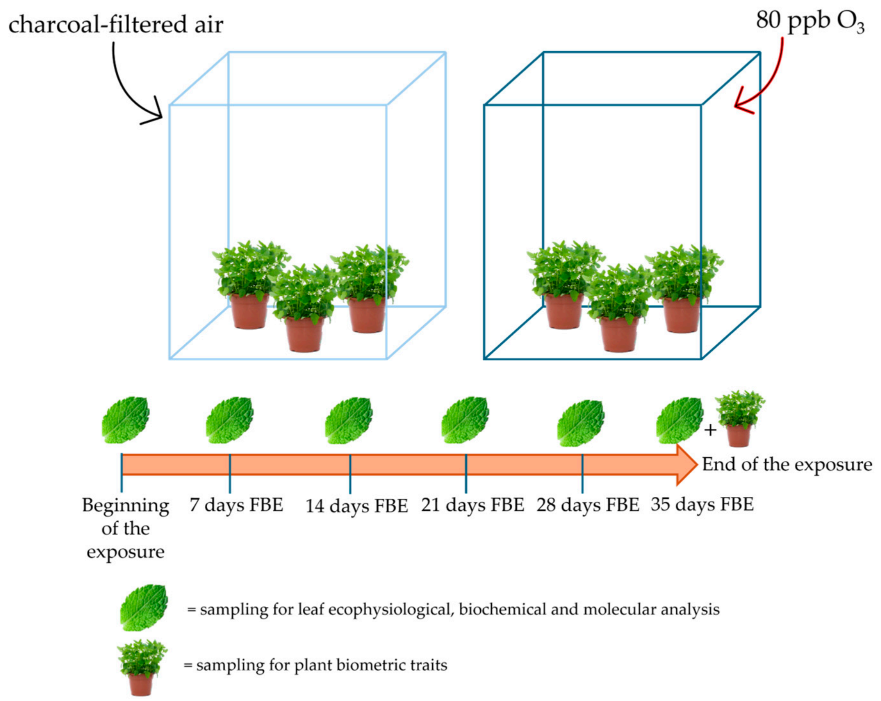

Figure S3. Visual summary of the experimental setup. Abbreviations: FBE, from the beginning of the exposure
